# Treatment of Xanthoma disseminatum – a systematic literature review

**DOI:** 10.1111/ddg.15824

**Published:** 2025-08-08

**Authors:** Inga Hansen‐Abeck, Mina Hillemans, Finn Abeck, Stefan W. Schneider, Nina Booken

**Affiliations:** ^1^ Department of Dermatology and Venereology University Hospital Hamburg‐Eppendorf Hamburg Germany

**Keywords:** non‐Langerhans cell histiocytosis, therapy, Xanthoma disseminatum

## Abstract

Xanthoma disseminatum is a rare disease from the spectrum of non‐Langerhans cell histiocytoses, which can be categorized into three types and is sometimes associated with systemic involvement. Due to the its rarity, there are no standardized treatment guidelines for this disease, making treatment in everyday clinical practice more difficult.

The aim of this study was therefore to summarize the therapeutic experience of the last 22 years by conducting a systematic literature review and thus generate an overview of the various treatment options. A systematic literature review on the topic of xanthoma disseminatum and applied treatment strategies was conducted in the PubMed/MEDLINE database for the period from 26 June 2002 to 26 June 2024.

The literature search resulted in 38 publications, 19 of which were included in the review. The therapeutic approaches of the studies analyzed can be divided into immunosuppressive, cytostatic‐based, lipid‐lowering, surgical, UV radiation, and laser‐based therapeutic approaches. Therapy with cladribine was reported most frequently. The current data is mainly based on case reports and case series, which are presented and summarized in this review. It can therefore be used as a basis for treatment decisions.

## INTRODUCTION

Xanthoma disseminatum (XD) is a rare disease first described by Montgomery and Osterberg in 1938. It belongs to the benign non‐Langerhans cell histiocytoses.^1^, ^2^, ^3^ The age at disease onset ranges from 8 months to 85 years. Usually, the disease develops in early adulthood and men are more often affected than women.^2^, ^4^, ^5^ The disease is characterized by proliferation of histiocytic cells for unknown reasons and secondary lipid depositions in dermis and internal organs.^1^, ^2^, ^6^

**FIGURE 1:**
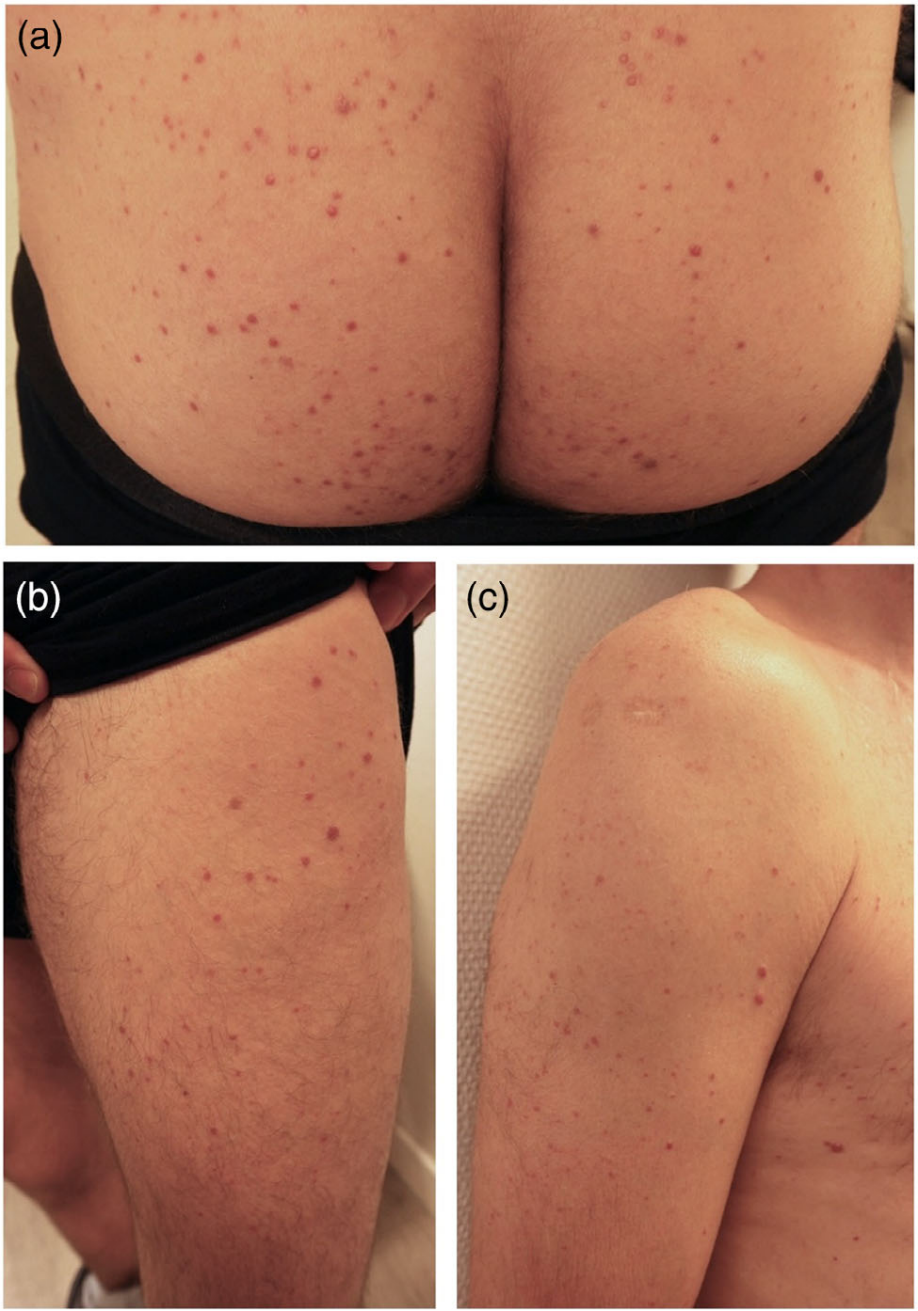
Clinical picture of a xanthoma disseminatum with disseminated, diffusely distributed reddish‐brown papules (a) gluteal (b) on the leg and (c) on the arm

Clinically, three forms of manifestation of XD are distinguished: a self‐limiting form, a persistent, purely cutaneous form, and the progressive systemic form with organ dysfunction and potential involvement of the central nervous system (CNS).^4^ The cutaneous manifestations present as an insidious onset of disseminated, cutaneous, yellowish‐red or reddish‐brownish papules (Figure 1).^7^ Eyelids as well as intertriginous areas and flexures of the extremities are characteristic localizations.^2^, ^8^ During the disease course, the lesions often disseminate and sometimes coalesce into plaques.^9^ Involvement of mucous membranes occurs in approximately 30–40 % of cases. Given that oropharynx, larynx, and conjunctivae are often affected, this may result in dysphagia, obstruction of the respiratory tract, and blindness.^7^ In 40 % of cases, the progressive systemic form is accompanied by diabetes insipidus (DI), which is caused by alterations in the hypothalamus‐pituitary region and is often irreversible.^7^

**FIGURE 2:**
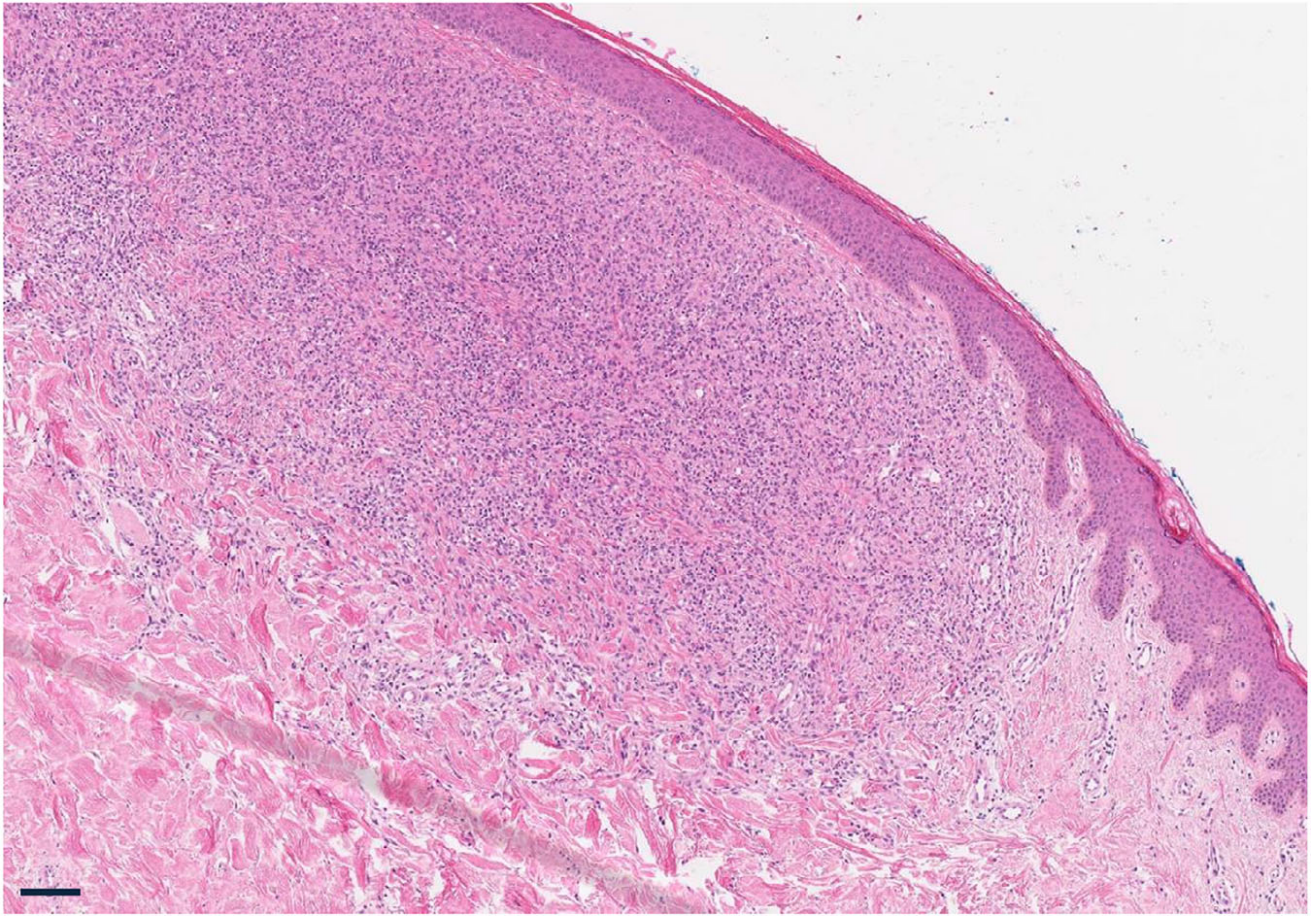
Histological picture: Epidermis with flattened rete ridges, dense infiltrates of macrophages and lymphocytes with few eosinophils (hematoxylin‐eosin stain, scale bar: 250 µm).

Histologic and immunohistochemical examinations are required for the diagnosis of XD.^10^ In histopathology, the initial stage presents with infiltration of macrophages with a dendritic form, with only few foam cells, lymphocytes, and eosinophilic granulocytes (Figure 2).^7^ In the later stages of the disease, xanthomatized Touton‐type macrophages with polynuclear giant cells extensively infiltrating the dermis usually dominate.^7^ Immunohistochemically, the surface markers CD68 and factor XIIIa of macrophages are important.^6^, ^7^ The markers S‐100, CD1a, and Birbeck granules are usually negative, facilitating the differentiation form Langerhans cell histiocytoses.^6^, ^7^ Computed tomography of the chest, magnetic resonance imaging of the head, laryngoscopy, and ophthalmic examinations may be performed to exclude potential systemic involvements.^8^, ^11^, ^12^

Given that there are no treatment standards for XD, it was the aim of this study to summarize the published therapeutic approaches in a systematic manner.

## METHODS

We performed a systematic literature search with the search terms “(xanthoma disseminatum) [title/abstract] AND (therapy) [all fields]”. The literature search was conducted in the database PubMed/MEDLINE for the period from 26 June 2002 to 26 June 2024. Subsequently, the hits were documented in a results table in Microsoft Excel. In a first screening round, the results were analyzed based on their title and abstract. Inappropriate results were rejected based on predefined inclusion and exclusion criteria (Table 1). In the second round, the remaining hits were examined based on their full text. Subsequently, the relevant information was extracted from the included publications and documented in a second results table. The screening was performed independently by two reviewers (M.Z. and I.H.‐A.) followed by a discussion of potential discrepancies. Moreover, the references in the included articles were screened for additional, relevant studies. The study was conducted according to the principles of *Preferred Reporting Items for Systematic Reviews and Meta‐Analyses* (PRISMA).

**TABLE 1:** Inclusion and exclusion criteria.

Inclusion criteria	Exclusion criteria
***Screening of title and abstract***	
Article examines relationship between XD and a therapy	Inappropriate topic Special forms of XD
Publication period: 26 May 2002 to 26 May 2022	Inappropriate methodology
Language: German or English	Disease onset before the age of 18 years
***Full text screening***	
	No cutaneous manifestations or therapy described No full text available or requestable
	Not in German or English language

## RESULTS

**FIGURE 3:**
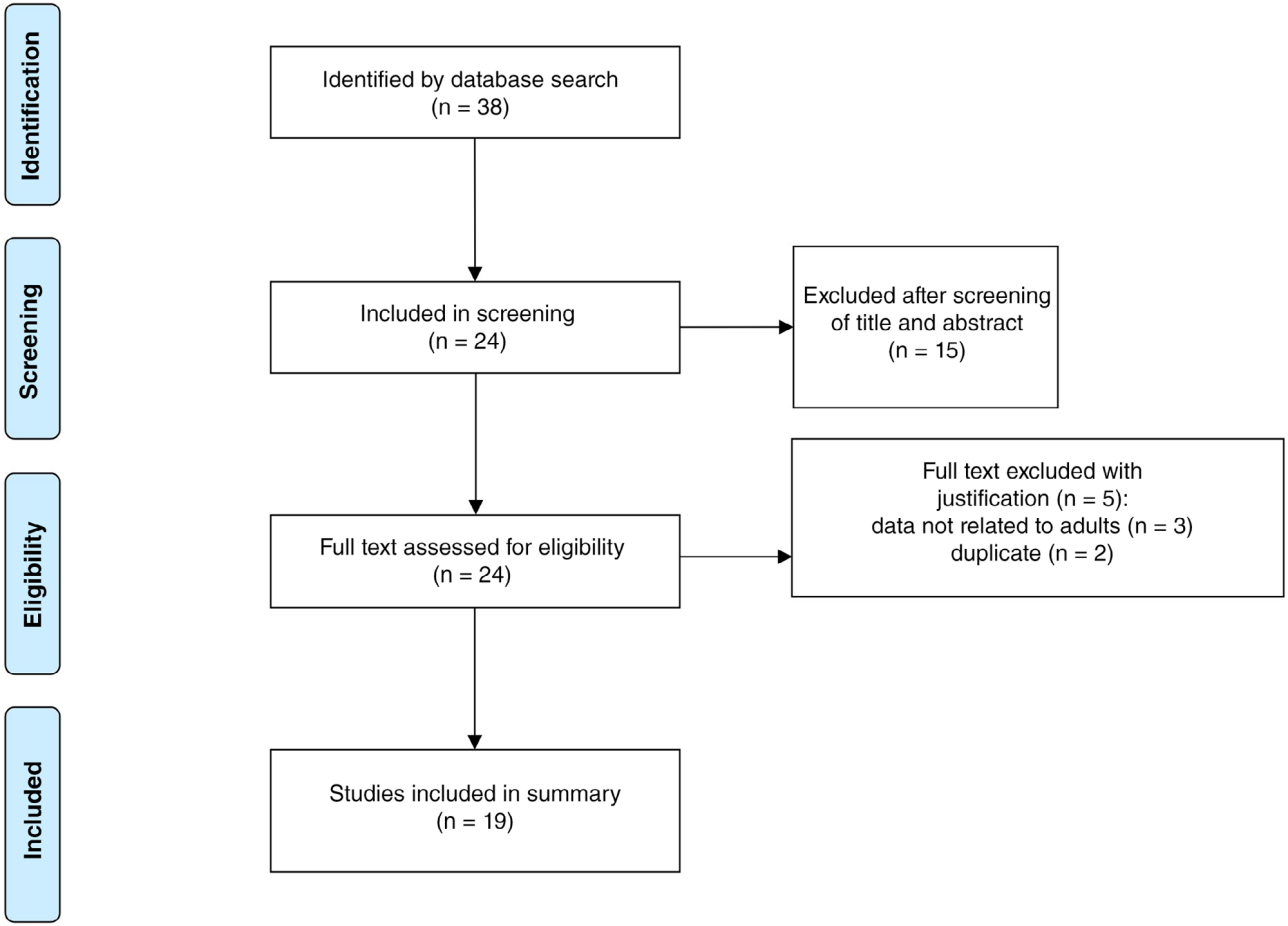
PRISMA flow chart.

The systematic literature search resulted in 39 articles in PubMed/MEDLINE. Altogether 15 publications could be excluded in the first screening round (Figure 3). In the second round, the remaining 24 publications were reviewed for their eligibility based on their full text. In this round, four additional studies were excluded. Accordingly, 20 studies reporting on 25 patients with XD were included in the analysis. An overview of the results obtained from the included publications is presented in Table 2.

**TABLE 2:** Table of results of the included studies on xanthoma disseminatum.

First author, year of publication, *methodology*	Clinical picturee and patient characteristics	Therapeutic approach	Result
Gayed et al., 2024 *Case report*	**Persistent XD, m, 65 years** *Cutaneous manifestation*: ‐ Lesion: red‐brownish papules, partly coalescing into plaques ‐ Localization: face, trunk, upper extremity, axillae	*Cladribine*: 6 cycles, 0.14 mg/kg/day for 5 days a month	Nearly complete remission of cutaneous manifestations
Jung et al., 2024 *Case report*	**Progressive systemic XD, m** *Cutaneous manifestation*: ‐ Lesion: brownish papules and plaques ‐ Localization: face excluding the nose; isolated in intertriginous areas *Systemic involvement*: DI	*Pulsed dye laser*: ‐ Initially: 0.45 ms, 7 mm, 7 J/cm2, 1 pass, 1 x per month ‐ Later: up to 0.45 ms, 10 mm, 10 J/cm2, 1 pass, up to 5 x per month *Vasopressin (DI)*	Good partial remission: cutaneous manifestations after 6 months of treatment
Zhou et al., 2023 *Case series*	**Progressive systemic XD, m, 20 years** *Cutaneous manifestation*: ‐ Lesion: brownish papules and plaques ‐ Localization: face including eyelids, intertriginous areas, trunk, genital region, thighs, popliteal fossae, oral mucosa *Systemic involvement*: dyspnea	*Cladribine*: 6 cycles, 0.14 mg/kg/day for 5 days a month	*Mucocutaneous manifestation*: partial remission (> 70 %) *Systemic involvement*: partial remission
	**Progressive systemic XD, f, 47 years** *Cutaneous manifestation*: ‐ Lesion: brownish papules and plaques ‐ Localization: face including eyelids, intertriginous areas, thighs, antecubital fossae, popliteal fossae, oral mucosa *Systemic involvement*: dyspnea	*Cladribine*: 5 cycles, 0.14 mg/kg/day for 5 days a month	*Mucocutaneous manifestation*: partial remission (> 80 %) *Systemic involvement*: complete remission
García‐Legaz et al., 2021 *Case report*	**Persistent XD, f, 40 years** *Cutaneous manifestation*: ‐ Lesion: multiple, rapidly progressive, yellow‐brownish papules ‐ Localization: face, upper extremities ‐ Symptoms: pruritus	*Narrowband UV‐B phototherapy*: (3 days/week): 54 irradiations, cumulative dose: 62.14 J/cm^2^	Partial remission, rapid alleviation of pruritus, no side effects
Tuan et al., 2019 *Case series*	**Progressive systemic XD, m, 34 years** *Cutaneous manifestation*: ‐ Lesion: yellowish papules and nodes ‐ Localization: face including eyelids, intertriginous areas, genital region, perianal region, oral mucosa *Systemic involvement*: DI	*Cladribine*: 5–9 cycles, 0.14 mg/kg/day for 5 days a month	*Mucocutaneous manifestation*: remission *Systemic involvement*: not specified
	**Progressive systemic XD, m, 35 years** *Cutaneous manifestation*: ‐ Lesion: yellowish papules and nodes ‐ Localization: face including eyelids, intertriginous areas, genital region, perianal region, oral mucosa *Systemic involvement*: DI		*Mucocutaneous manifestation*: remission *Systemic involvement*: not specified
Al‐Tarcheh et al., 2019 *Case report*	**Progressive systemic XD, f, 24 years** *Cutaneous manifestation*: ‐ Lesion: yellow‐brown papules, diameter: 1–7 mm ‐ Localization: face including eyelids, trunk, axillae *Systemic involvement*: arthralgia, DI, lungs (centrilobular emphysema)	*Cladribine*: 6 cycles, 0.14 mg/kg/day for 5 days a month *Desmopressin (DI)*	*Remission*: respiratory tract, arthralgia *Cutaneous manifestation*: stabilization
Sawatkar et al., 2016 *Case report*	**Progressive systemic XD, m, N/A** *Cutaneous manifestation*: ‐ Lesion: multiple, skin‐colored to yellowish papules and nodes, partly coalescing into plaques ‐ Localization: face, intertriginous areas, oral mucosa *Systemic involvement*: DI	*Imatinib*: ‐ initially 100 mg/day (oral) ‐ progressive increase to 400 mg/day	*Remission*: mucocutaneous manifestations, DI
Gupta et al., 2015 *Case report*	**Progressive systemic XD, m, 23 years** *Cutaneous manifestation*: ‐ Lesion: yellowish‐brownish papules, partly coalescing into plaques, brown macules ‐ Localization: face, chin, neck, axillae *Systemic involvement*: DI	*Cladribine*: 8 cycles, 0.14 mg/kg/day for 5 days a month *Desmopressin (DI)*	*Remission*: all cutaneous manifestations
Campero et al., 2016 *Case report*	**Progressive systemic XD, m, 32 years** *Cutaneous manifestation*: ‐ Lesion: disseminated, yellow‐brownish papules ‐ Localization: eyelids, trunk, axillae *Systemic involvement*: ‐ CNS: panhypopituitarism with DI and oligozoospermia as well as temporal lobe epilepsy	*Radiotherapy*: temporal lobe and pituitary: 38 Gy total dose*Anakinra*: ‐ initially 100 mg s.c./day for 15 months ‐ then 100 mg s.c./week for 24 months ‐ then 100 mg s.c./month for 12 months *Anticonvulsant therapy* (epilepsy)	*Radiation*: without effect *Anakinra*: remission of cutaneous manifestation and CNS involvement (on continuing anticonvulsant therapy)
Hsu et al., 2014 *Case report*	**Persistent XD, m, 70 years** *Cutaneous manifestation*: ‐ Lesion: nodules ‐ Localization: face, oral mucosa, intertriginous areas, genital region, medial thighs	*Excision* *Non‐ablative 1,450 nm diode laser*: 3 laser treatments of the face at 14‐day intervals over a period of 6 weeks, single dose: 18 J/cm^2^ x 25 ms	*Excision*: recurrence *Non‐ablative 1,450 nm diode laser*: remission of cutaneous manifestations on the face
Park et al., 2014 Case report	**Self‐limiting XD, m, 61 years** *Cutaneous manifestation*: ‐ Lesion: multiple, round to oval, pink‐brownish papules, diameter: up to 0.5 cm ‐ Localization: trunk, upper extremities	*Doxycycline*: 220 mg/day for 1 month *Ciclosporin*: 300 mg/day for 6 weeks	*Doxycycline und Ciclosporin*: no change During the course, self‐limiting
Mahajan et al., 2013 *Case report*	**Persistent XD, m, 18 years** *Cutaneous manifestation*: ‐ Lesion: multiple, grouped, orange‐brown, papulonodular lesions with smooth surface ‐ Localization: face including eyelids, neck, axillae, antecubital fossae, popliteal fossae	*Azathioprine*: 50 mg, 2 x daily In combination with *Prednisolone*: 40 mg every 2 days	*Cutaneous manifestations: s*tabilization, partly remission
Lee et al. 2011 *Case report*	**Progressive systemic XD, f, 63 years** *Cutaneous manifestation*: ‐ Lesion: yellow‐brownish pea‐sized papules; in some areas, confluent yellowish plaques ‐ Localization: face including eyelids, tongue, chest, neck, intertriginous areas, genital region, antecubital fossae, popliteal fossae *Systemic involvement*: urinary retention, dyspnea, and dysphagia	*Cyclophosphamide*: ‐ 50–100 mg daily for 2 years *Combination therapy (4 months)*: ‐ rosiglitazone (4 mg/day) ‐ simvastatin (10 mg/day) ‐ acipimox (500 mg/day) *Later simvastatin monotherapy*:10 mg/day for 5 months	*Cyclophosphamide*: no response *Lipid‐lowering agents*: remission of all skin lesions, dyspnea, dysphagia, and urinary retention
Khezri et al., 2010 *Case series with 8 patients; of these, 5 received treatment and were included in this review*.	**(1) Persistent XD, m, 55 years** *Cutaneous manifestation*: ‐ Lesion: yellow‐orange papules and plaques ‐ Localization: eyelids, trunk, proximal extremities	*Cladribine*: 8 cycles 0.14 mg/kg/BW i.v., for 5 days, one cycle/month	Complete remission
	**(2) Persistent XD, m, 46 years** *Cutaneous manifestation*: ‐ Lesion: yellowish papules ‐ Localization: face including eyelids, trunk	*Cladribine*: 5 cycles 0.14 mg/kg/day for 5 days a month	Nearly complete remission
	**(3) Persistent XD, m, 41 years** *Cutaneous manifestation*: ‐ Lesion: yellow‐orange plaques ‐ Localization: scalp, face including eyelids, trunk, axillae, upper extremities	*Cladribine*: 6 cycles 0.14 mg/kg/day for 5 days a month	Complete remission
	**(4) Progressive systemic XD, m, 67 years** *Cutaneous manifestation*: ‐ Lesion: reddish papules and nodes ‐ Localization: scalp, face including eyelids, trunk, intertriginous areas, perianal region *Systemic involvement*: DI, respiratory tract	*Cladribine*: 4 cycles 0.14 mg/kg/day for 5 days a month	Partial remission of cutaneous and systemic manifestations
	**(5) Persistent XD, m, 46 years** *Cutaneous manifestation*: ‐ Lesion: red‐brownish papules and nodes ‐ Localization: eyelids, axillae	*Cladribine*: 5 cycles 0.14 mg/kg/day for 5 days a month	High partial remission (approx. 80 %)
Kim et al., 2010 *Case report*	**Persistent XD, f, 47 years** *Cutaneous manifestation*: ‐ Lesion: yellow‐brown nodules and papules, partly coalescing into plaques ‐ Localization: scalp, face including eyelids, oral and nasal mucosa, neck, shoulders, axillae, perianal region	*Combination therapy (sequentially)*: ‐ surgical removal ‐ CO2 laser ‐ prednisolone oral (20–40 mg/day for 11 weeks, tapering over 10 weeks)	Progression of mucocutaneous manifestations
*Oka et al., 2010* *Case report*	**Progressive systemic XD, m, 30 years** *Cutaneous manifestation*: ‐ Lesion: multiple, yellow‐brownish papules, partly coalescing into plaques ‐ Localization: face including eyelids, tongue, neck, intertriginous areas, genital region, abdomen, antecubital fossae, popliteal fossae ‐ Symptoms: pruritus *Systemic involvement*: DI, upper respiratory tract, conjunctivae	*Initially, prednisolone monotherapy*:50 mg/day for 14 days, then 40 mg/day for 14 days and 30 mg/day for 1 month*Combination therapy (sequentially)*: ‐ clofibrate 1,500 mg/day + prednisolone 25 mg/day for 1 month ‐ clofibrate + dexamethasone, initially, 3 mg/day, for 5 months ‐ clofibrate + dexamethasone + etretinate 50 mg/day for 1 month ‐ dexamethasone 0.25 mg/day monotherapy	Progression of all manifestations *Clofibrate + dexamethasone + etretinate*: remission on tongue, stabilization of cutaneous manifestations, progression of CNS manifestations
Yusuf et al., 2009 *Case report*	**Progressive systemic XD, f, 32 years** *Cutaneous manifestation*: ‐ Lesion: confluent papules, plaques ‐ Localization: face, trunk, extremities *Systemic involvement*: larynx and pharynx, polyuria, polydipsia, dysphonia	*Prednisolone*: ‐ 20 mg 2 x daily for 22 weeks	Partial remission of mucocutaneous manifestations, larynx, and pharynx No change of dysphonia
Eisendle et al., 2008 *Case report*	**Progressive systemic XD, m, 42 years** *Cutaneous manifestation*: ‐ Lesion: yellow‐brownish papules ‐ Localization: face including eyelids, intertriginous areas, antecubital fossae, perianal region, nasopharyngeal and oral mucosa *Systemic involvement*: pharynx, conjunctivae, osseous lesion (left forearm)	*EtoposideSurgical treatment*: nostrils, wrist, oral mucosa*Interferon‐γ*: s.c., 2 million IU, 3 x/week for 3 months*Combination therapy*: ‐ rosiglitazone: 4 mg 1 x/day ‐ simvastatin: 10 mg 1 x/day ‐ acipimox: 250 mg 2 x/day	*Etoposide*: no change *Surgery*: remission of operated manifestations *Interferon*: remission of cutaneous manifestations, recurrence after discontinuation *Combination therapy*: partial remission of cutaneous manifestations, stabilization of mucosal and osseous manifestation
Hisanaga et al., 2004 *Case report*	**Progressive systemic XD, m, 68 years** *Cutaneous manifestation*: ‐ Lesion: reddish and yellow‐brownish plaques with a size of up to 15×20 cm and indurated margins, isolated papules ‐ Localization: back *Systemic involvement*: colon, rectum, lungs, DI	*Combination therapy*: ‐ systemic steroids ‐ antibiotics ‐ antimycotics	Patient died with respiratory insufficiency as a consequence of XD

### Physical treatment approaches

Surgical removal of cutaneous xanthomas as monotherapy or as complementary intervention to reduce the symptom load was described in several studies.^6^, ^12^, ^13^ In many cases, this approach could not prevent the development of new lesions.^6^, ^13^ Surgical interventions have also been described in the context of emergency indications, for example, to prevent obstruction of the respiratory tract in case of pharyngeal involvement.^12^

In three of the included studies, laser treatment of the face was performed.^6^, ^13^, ^14^ The use of 1,450 nm diode laser therapy in the affected facial areas achieved satisfactory cosmetic results and no recurrence or complications were described.^13^ In another case report, a CO_2_ laser was used for treatment. In this approach, however, recurrence was observed in the follow‐up at week 21.^6^ A recently published study reported on the successful use of a *pulsed dye* laser on the face.^14^

In one case, the pruritic component of persistent XD was alleviated by narrowband UV‐B phototherapy. Moreover, stabilization of the skin condition was also described.^15^

### Lipid‐lowering therapies

Lipid‐lowering therapies were used in three of the included case reports.^8^, ^12^, ^16^ In all of these cases, patients with pronounced manifestation of the progressive systemic variant of XD were treated.^8^, ^12^, ^16^ In two cases, a combination of several lipid‐lowering agents (rosiglitazone, simvastatin, and acipimox) was used, at least temporarily.^8^, ^12^ In contrast, Oka et al. combined the lipid‐lowering agent clofibrate with a systemic steroid therapy.^16^ In each case, the use of lipid‐lowering agents resulted in stabilization or regression of the condition.

### Immunosuppressive therapies

In a total of ten studies, various immunosuppressive therapies were described for both the persistent cutaneous and systemic variant of XD.^6^, ^12^, ^16^, ^17^, ^18^, ^19^, ^20^, ^21^

The use of systemic glucocorticoids, such as prednisolone or dexamethasone, has been widely reported for persistent and progressive forms of XD.^6^, ^16^, ^17^, ^18^ A tendency for remission was only described in combination with other immunosuppressive drugs.^17^ Apparently, their topical application alone also has no beneficial effect.^16^

In one case, the use of azathioprine in combination with prednisolone was described. This resulted in partial remission of the skin lesions during the three‐month observation period.^17^ In one case report, the calcineurin inhibitor ciclosporin had no effect on the skin condition.^21^

The interleukin‐1 receptor antagonist anakinra was used in one case of pronounced progressive systemic XD and resulted in remission of skin and CNS lesions.^19^ Interferon‐γ was also used in a patient with systemic XD.^12^ Remission of the skin lesions and stabilization of the systemic involvement were achieved for the duration of treatment, but disease progression was observed again within 3 months after drug discontinuation.^12^

### Cytostatic‐based therapies

Cytostatic‐based therapies were reported in altogether nine studies.^2^, ^8^, ^12^, ^22^, ^23^, ^24^, ^25^, ^26^, ^27^ In most cases, the patients suffered from progressive systemic XD.

With altogether twelve cases in six different studies, therapy with cladribine (Cd2‐A) was reported most often.^2^, ^22^, ^23^, ^25^, ^26^, ^27^ Seven patients suffered from the progressive systemic variant while the persistent cutaneous form was present in five cases. The patient age ranged from 20 to 67 years. All patients received a dose of 0.14 mg/kg body weight (BW)/day on five consecutive days per month for five to nine therapy cycles. In all reported cases, progression of the cutaneous lesions was observed to stop, with at least partial remission. Complete remissions were achieved in three of the twelve cases.^2^, ^4^ Remissions were also described in patients with mucosal lesions and CNS involvement.^2^, ^4^, ^27^ Progression of CNS involvement was prevented in all cases.^2^, ^22^, ^23^, ^27^

Treatment with the tyrosine kinase inhibitor imatinib significantly reduced the skin lesions of one patient.^24^ Given that the DI‐associated symptoms of the patient also decreased, this was interpreted as evidence for central remission of the disease.^24^

In one case report, cyclophosphamide treatment resulted in stabilization of the condition over a period of 2 years. There was, however, no evidence of remission.^8^ Etoposide was also able to halt disease dynamics without achieving remission of the existing symptoms.^12^

### Other treatment attempts

Administration of doxycycline for one month in persistent XD did not contribute to improvement of the skin condition.^21^ Oral administration of the retinoid etretinate in a patient with progressive systemic XD was also ineffective.^16^

## DISCUSSION

This systematic literature search revealed a heterogeneous data situation regarding the therapy of XD. Due to the rarity of the disease, a limited number of studies consisting of case reports and case series studies have been published worldwide. No prospective or retrospective trials have been published to date. This literature review can therefore serve as an important and useful basis for the treatment of patients with XD.

In its persistent form, XD is a purely dermatological disease. Given that spontaneous resolution is possible, a wait‐and‐see management approach may be warranted.^9^ In case of severe cosmetic impairment, laser treatment may be offered in addition to surgical removal. Given that these present purely ablative or symptomatic therapies, however, the risk of recurrence should be considered.^13^

In several case reports, the lipid‐lowering drugs simvastatin, rosiglitazone, acipimox, and clofibrate were effective even in progressive systemic XD.^8^, ^12^ This approach utilizes the indirect anti‐inflammatory potential of these agents, in particular. The mode of action is based on a reduction of the accumulation of proinflammatory lipids in foam cell‐like histiocytes.^12^ Given the comparatively favorable side effect profile, the use of lipid‐lowering drugs as first‐line therapy may be appropriate, if there are no contraindications. Based on the currently available data, it cannot be assessed conclusively whether the combination of several lipid‐lowering agents is required for successful treatment.

Interdisciplinary management is often required in patients with mucosal involvement and systemic manifestation. CNS involvement with development of DI is considered a potential complication.^2^, ^23^ In DI, the symptoms are usually sufficiently controlled by complementary treatment with desmopressin.^27^ Given that the resulting functional damage is considered irreversible, halting progression should be the primary objective.^23^ For the progressive systemic type of XD, successful use of Cd2‐A has been reported most frequently. ^2^, ^22^, ^23^, ^25^, ^26^, ^27^ This is a synthetic purine analog inhibiting DNA synthesis and repair in T and B lymphocytes.^28^ Cd2‐A is used, among other things, for treatment of myeloproliferative diseases. A presumed mode of action in XD is based on the similarity of monocytes and the histiocytes present in XD.^2^ Apart from sufficient symptom control on the skin, a decrease of mucosal involvement was also observed in some cases on Cd2‐A treatment.^2^, ^22^, ^23^, ^27^

In addition, the successful use of other cytostatic and immunosuppressive drugs has been published.^12^, ^19^, ^20^, ^24^, ^28^ The successful use of anakinra has been attributed to its anti‐inflammatory effect on macrophages and their proliferation.^19^ In general, these drugs should always be used after consideration of the risk‐benefit ratio, given that potentially severe side effects may occur. Comprehensive patient information and careful consideration of the therapeutic use depending on manifestation and symptoms should be mandatory. Moreover, it should be taken into account that these are successful reports of individual or a few cases. Based on the currently available data, however, the use of steroids, antibiotics, and retinoids does not appear to be advisable.^2^, ^22^, ^23^, ^27^

A potential limitation of this study with respect to completeness might be that the search was conducted in one database only. However, given that this is the most commonly used medical database, it can be assumed that nearly all relevant studies can be found here. It cannot be excluded that relevant studies were performed but remained unpublished (publication bias).

Additional studies on XD are urgently needed in order to develop evidence‐based treatment guidelines in the future.

## Conclusions

The present literature search provides an overview of the therapeutic approaches for xanthoma disseminatum published to date and may be used as a basis for treatment decisions in patient management. While surgical excision of xanthomas is accompanied by a high recurrence rate, it may be indicated in certain cases. This applies in particular to lesions causing functional impairment, such as obstruction of the respiratory tract. Lipid‐lowering agents should be considered as first‐line therapy if systemic treatment is required. With respect to other systemic therapeutics, the best evidence is currently available for Cd2‐A. In addition, the combination of various interventions may be considered for pronounced manifestations.

In summary, no specific recommendations can be formulated based on the currently available data. Nevertheless, treatment of patients with XD should be guided by the case reports and case series published to date.

## CONFLICT OF INTEREST STATEMENT

None.
